# Are we “Waiting for Godot”- A Metaphor for Covid-19

**DOI:** 10.1017/dmp.2020.280

**Published:** 2020-06

**Authors:** James J. James

W*aiting for Godot*, a play by Samuel Beckett, is representative of what has come to be known as the Theatre of the Absurd. Variously defined as existentialist, dystopic, post-modern among other genres, it is characterized by obscure themes, illusory hopes, awaiting an event that never occurs and lacking a conclusion in the dramatic sense. Without defining specific parallels, the overall effect on the theatregoer is all too close to that felt by one reviewing the evolution of COVID-19 and our responses to it. One parallel that is worth pointing out is the voluminous, often paradoxical, analytical and critical literature published on Beckett’s work in attempting to explain its meanings and underlying philosophy such that almost any characterization of the work can be supported. Similarly, with COVID-19 there is a plethora of published reports on every aspect of the disease that allows supporting almost any opinion or policy. One of the hallmarks of Beckett’s mature works was a minimalist approach. Likewise, I plan to use this approach in presenting a review of where we are with various aspects of COVID-19, and, more importantly, how we can no longer afford to await the arrival of Godot.

Godot in this case might well be the arrival of an effective vaccine but, as in the play, that might prove to be more an illusion of hope than a reality. The obstacles to fielding a vaccine anytime soon necessitate reassessing acceptable and effective interventions to at least slow the rate of transmission. What is especially troubling in depending on a vaccine as the answer to COVID-19 is that the highest risk group (the elderly) historically are most refractive to vaccines in general, plus fewer than 50% of Americans plan to accept a vaccine upon its release.^1^ Given these considerations we need to take another look at Herd Immunity (HI) and its role in this pandemic. Importantly, we are addressing HI as a naturally occurring phenomenon and not as an intervention that would purposefully expose individuals to a potentially serious disease. Just as important, the HI level should not be looked at as a static number nor as an all or none state. Rather, as the overall level of population immunity increases the optimal HI level is approached and the rate of transmission decreases, resulting in a “flattening of the curve”. The optimal level of HI for COVID-19 is unknown, with various estimates ranging from the low 20s to as high as 90% to eliminate transmission. If the lower estimates are indeed correct then we may well be seeing an HI effect in the New York City area where the curve for new test positives has remained flat throughout the recent increases seen in most other states.^2^

As of this morning (July 29, 2020) there have been a reported 17,025,617 “cases” of COVID-19 and 665,947 deaths globally with 4,498,887 cases and 152,358 deaths recorded in the US. These are large and alarming numbers when reported as unadjusted numerators but when presented in terms of overall population rates, they are far less frightening. For the US the reported “case” rate is 1.3% and the mortality risk is .045% with the corresponding global figures approximately one sixth of those for the US (https://www.worldometers.info/coronavirus/). The term case is placed in quotation marks above because it is misleading. Reported, cases are, for the most part, a positive lab test and not the presence of disease or injury, which is the proper definition of a medical case. With COVID-19 this is more than semantics as the best case CDC estimate of asymptomatic positives is 40%^3^ and the overall population case rate, by inference, could be but 0.8%.

Another variable that we need to look at is testing, both its accuracy and the resulting ratio of true to total positives. The accuracy is dependent on a test’s sensitivity and specificity, which can only be estimated for COVID-19 as there is no true gold standard to measure against given that we are dealing with a new disease. A further complication is that there are well over 50 different tests being used across multiple testing and reporting protocols with varying degrees of accuracy. This is not to negate in any way the great importance of testing in the diagnosis, understanding and control of COVID-19, but to underscore that it is not perfect and may well contribute to the flawed value of case reports as an indicator of the health impacts of the pandemic. Just one example to highlight this point—for the US population, assuming an overall COVID-19 prevalence of 2%, using a test with 99% sensitivity and specificity will result in a 33% false positive rate. Such a result, if even in the ballpark, would further significantly reduce the estimated population medical case rate of 0.8% given above.

A final consideration demonstrating the irrationality of using positive tests as a yardstick for measuring the health impact of COVID-19 is the dramatic shift in the mean age of test positives from several months ago to the present time. One constant that has been evident through the course of this pandemic is that over 80 percent of fatal outcomes occur in those over 65. With the recent much publicized spike in US cases not enough attention has been paid to the shifting demographics of the test positives. Florida is most representative of what we are seeing nationwide where the average age of a test positive has gone from 54 in May to 35 today.^4^ This makes any comparisons of overall health impact over time absurd as current medical cases are simply not equivalent to earlier ones because of significant differences in average age. This is further underscored by the fourfold increase in testing we have seen since early May as the overall number of asymptomatic positives will increase along with the true cases, further diluting the medical significance of the recent spikes. The overall effect of these shifts is demonstrated by the fact that while there has been a fourfold increase in daily “cases” since June 1^st^ there has been a much more modest increase in mortality rates of just above 10% (https://www.nytimes.com/interactive/2020/us/coronavirus-us-cases.html).

The above discourse is not intended to diminish the seriousness of COVID-19 for those afflicted with it. Clinically it can be a devastating and too often lethal malady with profound impacts that affect patients, loved ones and the health systems caring for them. However, and most unfortunately, COVID-19 has become as much a divisive political issue as a medical one, making it difficult for individuals to identify accurate information from amongst conflicting opinions and advice. The tool that has been weaponized in these debates is statistics and it is interesting how a common set of data can be used to support opposing arguments and conclusions. Another victim of this politicization is sound and effective health communication that has unfortunately become the province of a biased media which has nurtured an all too effective campaign of fear. This fear has permeated our population and the discussion above is meant to provide a more realistic assessment of the real risk of serious illness from COVID-19 so that individuals can make more informed choices as to acceptable risk and authorities can better justify interventions and mandates. This, of course, is already beginning to happen as evidenced by younger individuals being more willing to congregate while, overall, the older age group rightly continues to adhere to sound social distancing practices. We could at this point discuss those practices and the current controversies, such as the use of face coverings, but these issues are receiving more than enough attention elsewhere and would be simply repetitive here. However, there is one raging controversy that demands our attention and that is concerning our school aged children.

From the beginning of our engagement with COVID-19, one consistent analogy used to define our interaction has been that we are at war with the virus. In many ways this military analogy has been obvious and helpful, but we have too often failed to recognize that in many ways we have also been at war with ourselves. The virus has certainly been a formidable foe with casualty counts enumerated above. But what of the casualties resulting from many of our ill-advised interventions, most notably the K-12 school closures affecting some 55 million children across the US.^5^ The full impact of the collective educational, economic, social, psychological, and physical damage to these children is yet to be tabulated but the sum total of healthy life-years in terms of morbidity and pre-mature mortality will more than likely far exceed that caused directly by the virus. The fact that this harm is inflicted on those virtually immune to serious medical outcomes secondary to COVID-19 is a self-inflicted tragedy. Why do we even have this situation? Firstly, we recognize that we are involved in a war but fail to accept the fact that war generates casualties; secondly, our operations to date have been tactical and defensive—we need an overarching strategy that includes accepting some degree of reasonable risk; and, thirdly, we need to preserve our strength going forward, and that is first and foremost our children. To continue school closures awaiting something that may not even arrive is clearly an illusion of hope and tantamount to fostering systemic child abuse.

“You’re on Earth. There’s no cure for that.”

– Samuel Beckett

## References

[1] Crist C. WebMD poll: most would wait on COVID vaccine. Published July 28, 2020. https://www.webmd.com/lung/news/20200728/webmd-covid-vaccine-poll?ecd=wnl_spr_072820_COVID&ctr=wnl-spr-072820-COVID_nsl-LeadModule_title&mb=cVOaDtCRn5byGX7BtG0fiBK5SPsKg1NYZq1z28gUo%404%3d. Accessed August 11, 2020.

[2] James JJ. COVID-19: yin and yang and herd immunity. Disaster Med Public Health Prep. 2020. doi: 10.1017/dmp.2020.229. PMC8060622 32631463

[3] Centers for Disease Control and Prevention. COVID-19 pandemic planning scenarios. Published July 10, 2020. https://www.cdc.gov/coronavirus/2019-ncov/hcp/planning-scenarios.html. Accessed August 11, 2020.

[4] Moser W. Why changing COVID-19 demographics in the US make death trends harder to understand. The COVID Tracking Project. Published June 26, 2020. https://covidtracking.com/blog/why-changing-covid-19-demographics-in-the-us-make-death-trends-harder-to. Accessed August 11, 2020.

[5] Sharfsten JM, Morphew CC. The urgency and challenge of opening K-12 schools in the fall of 2020. JAMA. 2020;324(2):133–134. doi: 10.1001/jama.2020.10175. 32478827

